# Editorial: Superficial fungal infections

**DOI:** 10.3389/fcimb.2023.1285771

**Published:** 2023-09-11

**Authors:** Suzana Otašević, Roderick Hay

**Affiliations:** ^1^ Department of Microbiology and immunology, University of Niš, Niš, Serbia; ^2^ Department of Dermatology, King’s College London, London, United Kingdom

**Keywords:** superficial fungal infection, mucosal fungal infection, candidosis, diagnostics, aspergillosis

Superficial fungal infections (SFI) represent a major health concern due to their high prevalence, affecting about 1.2 billion people worldwide (Denning and Bromley, 2015). Despite being traditionally associated with fungal infections of the skin, hair, and nails, this type of infection also includes fungal mucosal infections. More importantly, additional issues related to SFI are the occurrence of chronic, recurrent infections and emerging resistance to antifungal drugs. Numerous surveys have aimed to determine different aspects such as pathogenic properties of different fungal species, novel antifungal substances with different mechanisms of action, and new, rapid diagnostic methods.

Yeasts of the genus *Candida* (*C.)* are members of a healthy individual’s microbiota (Gulati and Nobile, 2016) and can asymptomatically colonize mucous membranes (oral, intestinal, vulvovaginal) and skin. They are found as part of the oral microbiota in approximately 75% of the population, and can colonize the tongue, buccal and palatal mucosa, restorative materials and dental prostheses (Vila et al., 2020). In most immunocompetent individuals, *C. albicans* is a commensal that co-exists in balance with other microorganisms of the microbiota, with established immunotolerance by the host’s immune syst (Gulati and Nobile, 2016). A disturbance of the local environment can cause an imbalance (changes occur due to a decrease in the local pH, which is mostly the result of poor dietary habits and hygiene), leading to the rapid reproduction of fungi and the onset of the infection (Gulati and Nobile, 2016). With this in mind, *Candida* species have been analyzed, with several studies aiming to uncover the yeast’s contribution to the development of oral microbiome related diseases, investigating the potential roles of *Candida* spp., either independently, or in cooperation with bacteria, in the pathogenesis of dental caries (Lemos et al., 2013). In this Research Topic in a study conducted by Cvanova et al., the authors suggested that species of the *Candida* genus, primarily *C. albicans* and *C. dubliniensis*, should be considered the “keystone pathogens” which are associated with dental caries in children, along with behavioral factors, the presence of cariogenic bacteria, as well as genetic predisposition.

Furthermore, another form of mucosal candidosis, vulvovaginal candidosis (VVC) - one of the most common infections affecting women, with prevalence rates ranging between 15% and 30%, remains a persistent health problem that has yet to be fully resolved both in theoretical and in practical terms (Sobel and Nyirjesy, 2021). The extremely high prevalence of VVC in pregnant women is a significant issue in neonatology, since the colonization of the birth canal with *Candida* species represents a major risk for neonatal infection. This infection can affect the pregnancy outcome, especially when cervical cerclage is performed, and can also be a substantial issue when *in vitro* fertilization is attempted (Ignjatović et al., 2020). Increased levels of 17-b estradiol (E2), due to pregnancy, use of hormonal contraceptives, or hormonal replacement therapy in postmenopausal women, have long been recognized as risk factors for yeast infections, along with the factors contributing to the increase in the virulence of fungi, such as: i) the expression of proteins essential for adhesion and invasion; ii) secretion of hydrolytic enzymes and iii) the morphogenetic transition of the fungus from yeast to hyphal form. The ability to transform into mycelial form is considered a major pathogenic characteristic of *C. albicans* species, since the mycelial growth form enables strong biofilm formation. It has already been established that E2 affects the growth of *C. albicans* (De Micheli et al., 2002). However, the current survey of Bataineh et al. demonstrated its role in the yeast-hyphal transition as well, and identified a regulatory gene complex mediating this morphogenesis. The results of this research are significant, considering the role of E2 in the development of fungal infection, and may serve as the foundation for the development of new therapeutic principles in the treatment of VVC.

Moreover, the rising resistance of *Candida* spp. to antifungal drugs influences ongoing studies aiming to identify new, effective antimycotics for local or systemic use, test the possible antifungal and antibiofilm properties of natural substances, or investigate possible synergistic effects of antifungal medications and these natural substances (Donders et al., 2022; Tran et al., 2022). Herein published, a particularly intriguing manuscript of Hu et al. investigated *in vitro*, and more interestingly, *in vivo* antifungal mechanisms of the action of sanguinarine (SAN) isolated from *Macleya (M.) cordata*. Previous research has reported that *M. cordata* alkaloids exhibit antifungal effects against *Trichophyton rubrum*, and inhibitory effects on the formation of *C. albicans* biofilms (Yang et al., 2012; Zhong et al., 2017). Subsequently, in the manuscript of Hu et al., it was demonstrated that the *in vivo* findings support the antifungal efficacy of SAN observed *in vitro*, showing that SAN is able to reduce pathogenic *C. albicans* cells within the deep organs, implying the potential for it to be considered in the treatment of candidosis, following additional research.

In addition to invasive candidosis, another challenge for the design and implementation of new treatment strategies is invasive aspergillosis (IA). Considering that IA is a fungal infection with very high mortality (Ledoux et al., 2020), and the increasing antimycotic resistance of *Aspergillus* spp., study results of Li et al., published in this Research Topic, offer encouraging insights regarding the determination of novel antifungal targets, as well as pathogenic mechanisms of *Aspergillus* spp. Since there is an abundance of histone posttranslational modifications observed in *Aspergillus* (Zhao et al., 2011), which could impact fungal growth, virulence and more importantly, antifungal drug sensitivity, this review highlights the importance of understanding the effects of different histone posttranslational modifications in the development of new strategies for the prevention and management of fungal infections.

In addition to investigating virulence factors and potential new therapeutic strategies, research efforts are also focused on the development of innovative techniques for detecting and identifying fungi, as well as diagnosing fungal infections. Since the conventional procedures such as culture for the diagnosis of SFI is both time consuming and relies on expert knowledge, development of new, non-culture methods, is of a great importance. Recent and ongoing research, published in this Research Topic, introduced a new imaging tool, dynamic full-field optical coherence tomography (D-FF-OCT), that can be used for the visualization and differentiation of microscopic filamentous fungi, facilitating the identification of moulds, and thus accelerating the diagnostic procedure (Maldiney et al. ).

## Author contributions

SO: Writing – original draft. RH: Writing – original draft.
